# Belatacept inhibit human B cell germinal center development in immunodeficient mice

**DOI:** 10.1038/s41598-023-40700-w

**Published:** 2023-08-24

**Authors:** Chloé Samson, Allan Thiolat, Anissa Moktefi, José L. Cohen, Caroline Pilon, Philippe Grimbert

**Affiliations:** 1 Université Paris-Est, UMR_U955, UPEC, 94000 Créteil, France; 2 Inserm, U955, 94000 Créteil, France; 3 Groupe Hospitalo-Universitaire Chenevier Mondor, Service d’Anatomopathologie Clinique, Assistance Publique-Hôpitaux de Paris (AP-HP), 94000 Créteil, France; 4 Groupe Hospitalo-Universitaire Chenevier Mondor, Centre d’Investigation Clinique Biothérapie, Fédération Hospitalo-Universitaire TRUE, Assistance Publique-Hôpitaux de Paris (AP-HP), 94000 Créteil, France; 5 Groupe Hospitalo-Universitaire Chenevier Mondor, Service de Néphrologie-Transplantation, AP-HP, 94000 Créteil, France

**Keywords:** Allotransplantation, B cells

## Abstract

The humoral response mediated by alloantibodies directed against donor HLA molecules (DSAs) is one of the main causes of graft loss in kidney transplantation. Understanding the pathophysiology leading to humoral kidney rejection as the development of therapeutic tools is therefore a main objective in the field of solid organ transplantation and necessitate adapted experimental models. Among the immunosuppressive agents used in renal transplantation, belatacept, a fusion protein targeting T costimulatory molecules has shown its ability to prevent more efficiently the secretion of DSA by different mechanisms including a direct action on plasma cells but also on B lymphocytes and follicular helper T lymphocytes (Tfh) cooperation. This cellular cooperation occurs within germinal centers (GC), the seat of B lymphocytes differentiation. Here, we aimed to develop a dedicated mouse model in which human GC would be functional to study the effect of belatacept on GC formation and the ability of B lymphocytes to secrete immunoglobulin. We next demonstrate that belatacept inhibits the formation of these GCs, by inhibiting the frequency of Tfh and B lymphocytes. This alters the B maturation and therefore the generation of plasma cells and consequently, immunoglobulin secretion.

## Introduction

Kidney transplantation remained the treatment of choice for end-stage chronic kidney disease, but long-term survival improvement is stagnating^1–3^. This observation is mainly related to the occurrence of antibody-mediated rejection (ABMR) which remains the death-censored leading cause of transplant loss across all solid organ transplants^4^. Anti-HLA donor-specific antibodies (DSAs) arising after kidney transplantation, also called de novo donor-specific antibodies (*dn*DSAs), have increasingly been recognized as the leading cause of both acute and chronic ABMR^5^. Approximately 15–30% of kidney transplant recipients develop *dn*DSAs^6^.

Belatacept (cytotoxic T lymphocyte–associated antigen 4 [CTLA4]-Ig; LEA29Y; Bristol Myers Squibb) is a human fusion protein combining the extracellular portion of CTLA-4 that has been mutated to confer greater binding avidity to CD80 and CD86 and the constant region fragment of human IgG1. CTLA-4 binds to surface costimulatory ligands of antigen-presenting cells (APCs) and in a lesser extent on T cells, and thus, prevents their interaction with CD28, thereby blocking T cell activation^7,8^.

Long-term follow-up analysis of phase 3 clinical trial using belatacept showed that recipient mortality and the graft failure rate at 7 years after transplantation were significantly lower in the group of recipients treated with belatacept compared with the control recipients treated with calcineurin inhibitors (CNIs). Interestingly, the incidence of *dn*DSAs at year 7 was significantly lower in belatacept-treated patients than in CNI-treated patients^9^. These clinical results remain to be explained by experimental studies aiming to analyze the effect of belatacept, as other costimulatory inhibitors molecules, on different steps of the B cell–mediated response in humans.

Using a preclinical model of ABMR after renal transplantation in macaques, Kim et al. showed that belatacept prevents the maturation of B cells in peripheral blood and memory T cell populations in secondary lymphoid organs and finally suppresses germinal center reconstruction after T cell depletion*.* However, in this non-human model, belatacept was used as additional immunosuppressive regimen including Steroids, Tacrolimus, monoclonal Anti-CD3 antibodies and Alefacept^10^. Our previous in vitro human studies showed that belatacept reduces plasmablast differentiation, Ig production, and expression of the major transcription factor involved in plasma cell function, Blimp-1, independently of T lymphocytes^11^. When Tfh and B lymphocytes are co-cultured, belatacept blocks CD28-mediated Tfh activation. Moreover, patients treated with belatacept, exhibited a reduced proportion of blood effector B lymphocytes, and activated Tfh (PD1^+^ ICOS^+^) compared to control transplant patients treated with CNI^11^. Taken all together, our results showed that belatacept modulates the function of B cells directly and at the level of the B cell-Tfh interaction.

Studying human B and T cells interaction in humanized mice is quite a challenge. B cells do not survive long time post infusion in classical humanized mice models. Some authors have developed models like the one of Jangalwe et al.^12^ but they are technically hardly achievable: irradiation, human fetal tissues and special NSG mice were used. Here, using an original, easily reproductible experimental in vivo model of human germinal centers formation in NSG (NOD/scid/IL-2Rγ-/-) mice, we demonstrate for the first time on human cells the detrimental and specific role of belatacept on human germinal centers formation as single immunosuppressive regimen.

## Results

### B cells enrichment in mice spleen using CD8 depleted PBMCs

**Figure 1:**
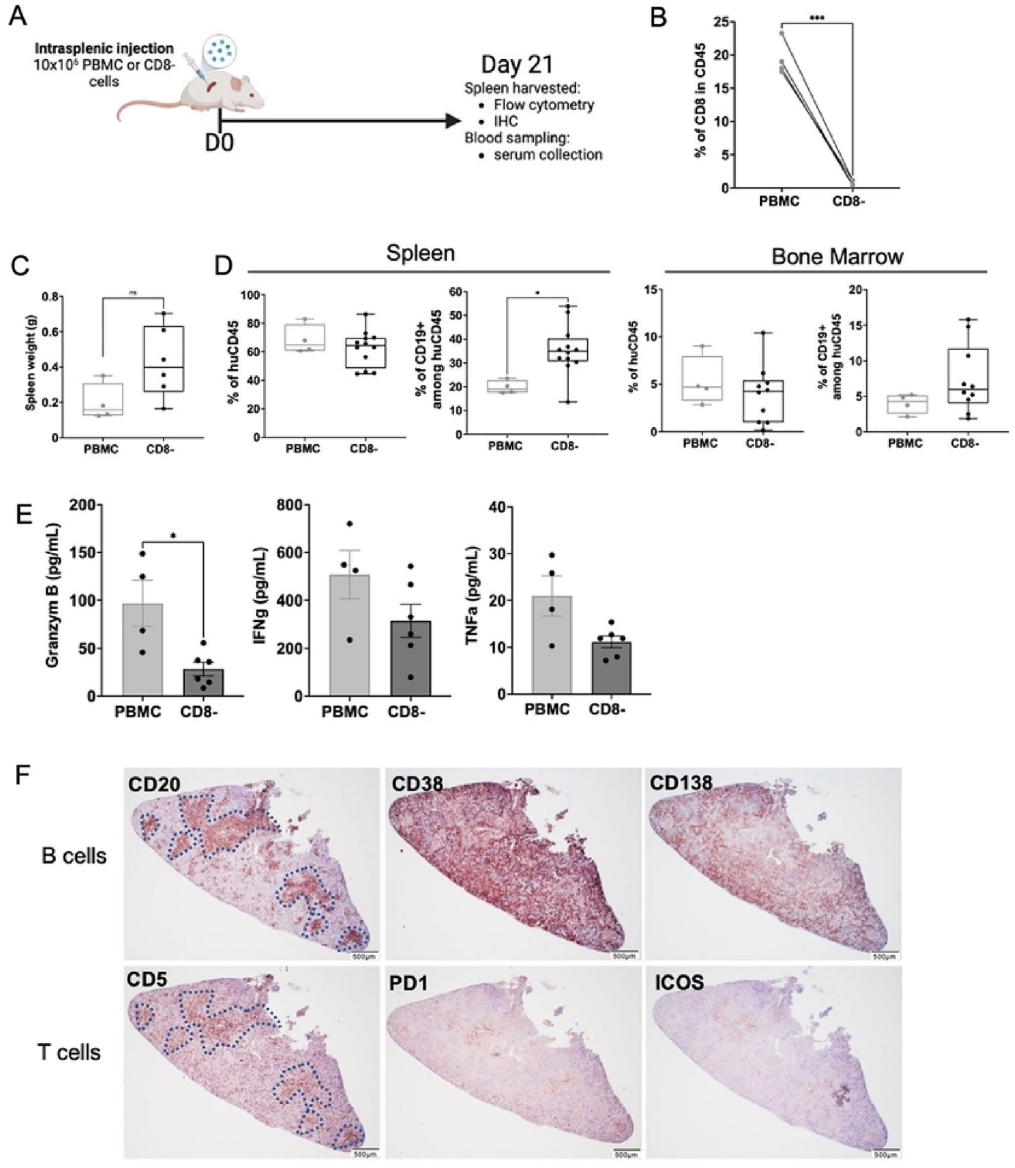
B cells engraftment after human CD8 depleted PBMCs infusion. Intrasplenic injection of NSG mice were realize with 10 × 10^6^ of PBMC or CD8 depleted PBMCs (CD8-). Mice were sacrificed at day 21 and spleen were harvested for analysis (**A**). (**B**) Proportion of CD8 in CD45 cells before and after negative selection of CD8. Statistical significance of CD8 proportion of before and after negative selection was determined using paired t-test, *** *p* ≤ 0.001. (**C**) Spleen weight (g) of both group at day 21. (**D**) Flow cytometry detection of human cells at day 21 in the spleen and bone marrow of NSG mice. Histograms represent percentage of human CD45 + and CD19 + cells are represented. All samples were initially gated for living lymphocytes by forward and side scatter. The cumulative data of five independent experiments are shown. Each point represents a single xenograft; data are presented as mean percentage ± SEM. (**E**) Granzyme-B, IFN-γ and TNF-α concentration in the mice serum at D21. Statistical significance from the controls (PBMC) was determined using Mann–Whitney tests. *p ≤ 0.05. (**F**) Serial sections were stained with anti-human CD20/CD38/CD138 for B cells analysis and anti-human CD5/ICOS/PD1 for T cells analysis. Original magnification × 20 for CD5 and CD20 staining, × 100 for CD38, CD138, PD1 and ICOS staining. Dotted line in CD20 and CD5 images represents T and B cells colocalisation.

Few in vivo models allow the study of human germinal centers and the B and T cells interaction. Then, to facilitate the B cells engraftment, we infuse human PBMC directly in the spleen of immunodeficient NSG mice by intrasplenic infusion. Since CD8 T cells seem to be directly implicated in xeno-GVHD clinical symptoms development, we compared infusion of total PBMC with an infusion of CD8-depleted PBMC (Fig. 1A). CD8 proportion after depletion was significantly reduced (Fig. 1B). On day 21, spleen weight with CD8-depleted PBMC were higher than in PBMC mice (Fig. 1C). On day 21, no difference was observed in total human cells reconstitution evaluated by the percentage of huCD45, between mice infused with PBMC or CD8- depleted human cells (Fig. 1D). However, CD8 depletion significantly favored B cells survival. The proportion of CD19 + among huCD45 cells increases from 19.83 to 35.48% in CD8-depleted injected mice. Human B cells were also detected in the bone marrow of the grafted mice (Fig. 1D). Cytokines production such as Granzyme-B, IFN-γ and TNF-α known to be implicated in xeno-GVHD was reduced (Fig. 1E). For these reasons, we decided to use CD8-depleted infusion for the next experiments to be able to follow the cell composition in vivo without being limited by potential mortality due to xeno-GVHD. We then analyzed the germinal center formation in the spleen by immunohistochemistry at D21. The comparison of hematoxylin and eosin staining of normal NSG mice with CD8-depeleted PBMC infused NSG spleen show difference in size and cells structure (Fig. S1). Immunohistochemical staining formally confirmed the presence of a B cell zone (Fig. 1F). Indeed, CD20 expression showed the naive B cells in the middle of the GC. CD38 expression showed the differentiated cells at the periphery of follicles whereas the CD138 positive cells outside of GCs showed the terminal stage of B cell differentiation which corresponds to plasma cells (Fig. 1F). The distribution of the different stages of maturation of B cells was thus hierarchically respected compared to a classic secondary lymphoid organ. The presence of the T zone was also evaluated by the CD5 + expression (Fig. 1F). Thus, the CD5 + expression in the NSG mice spleen highlights the presence of T cells. The joint expression of ICOS and PD1 suggests that these T cells may be Tfh (Fig. 1F). All, those results suggest the presence of functional human germinal centers in mice.

### Belatacept reduces B cells survival and human germinal centers formation

**Figure 2:**
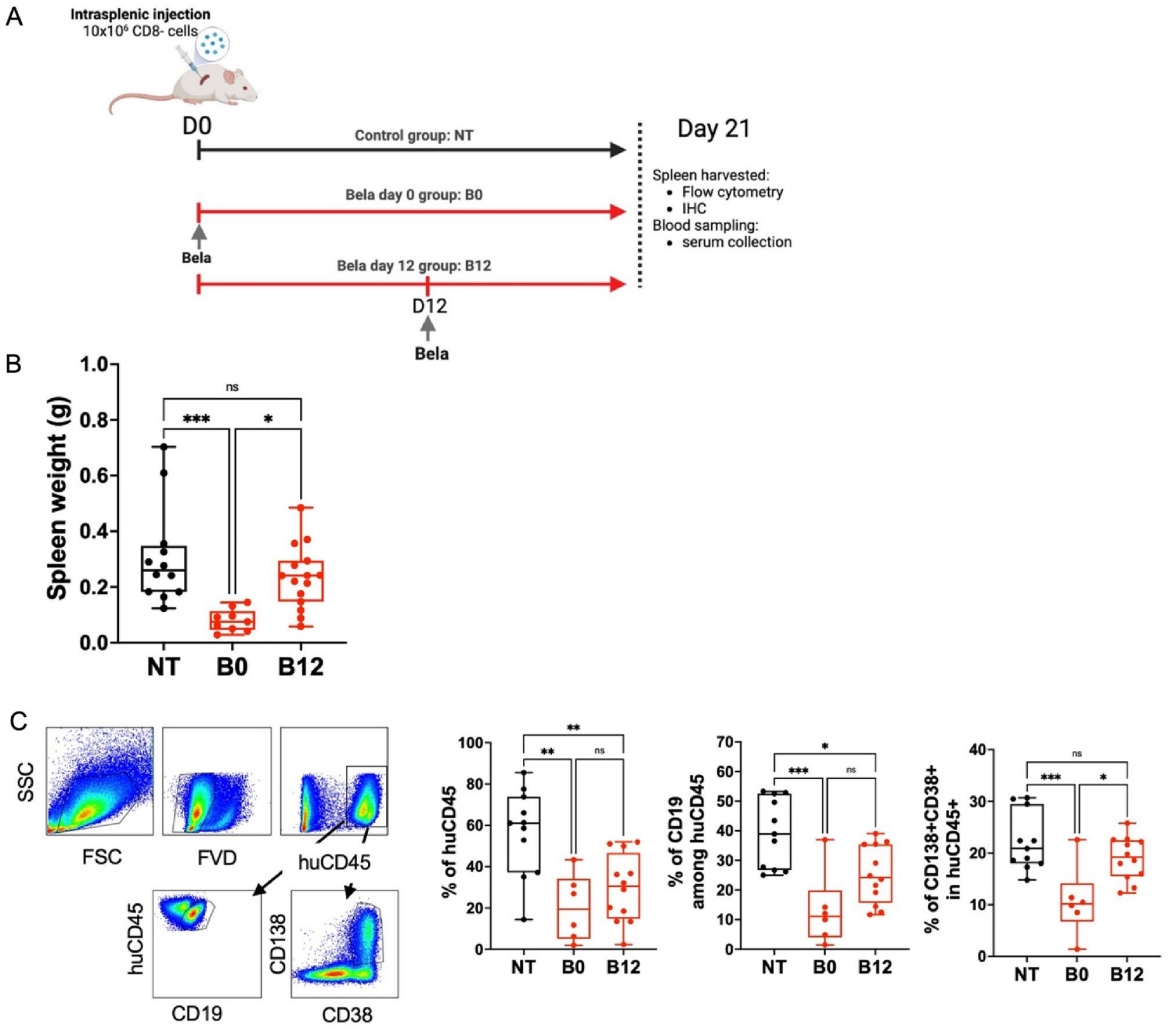
Belatacept decreased human B cells persistence. (**A**) Protocol design. Intrasplenic injection of NSG mice were realize with 10 × 10^6^ of CD8 depleted PBMCs (CD8-). Belatacept (5 mg/kg) were injected either at the same day of cells injection (B0) nor at day 12 (B12). Untreated mice (NT) served as control group. Mice were sacrificed at day 21 and spleens were harvested for flow cytometry analysis. (**B**) Spleen weight (g) of each group at day 21. The cumulative data of five independent experiments are shown. Each point represents a single xenograft; data are presented as mean percentage ± SEM. (**C**) Gating strategy and percentage of human CD45 + cells, CD19 + B cells, CD38 + CD138 + B cells in untreated (NT) and treated groups (B0, B12). The cumulative data of five independent experiments are shown. Each point represents a single xenograft; data are presented as mean percentage ± SEM. Statistical significance from the controls (NT) was determined using one-way anova and Tukey’s multiple comparisons test. *p* ≤ *0.05, **0.01, ***0.001.

To assess the effect of belatacept on B cells differentiation and on the GC formation in the spleen of NSG mice, we adopted two different strategies: early treatment on day 0 (B0) to study the effect on the structural establishment, and at day 12 (B12) to determine the effect of belatacept on existing functional germinal structures (Fig. 2A). Only day 0 treatment with belatacept (B0), significantly reduced the spleen weight and size compared to the untreated mice (Fig. 2B). Also, both belatacept treatments (B0 and B12) significantly decreased the percentage of huCD45 + cells in the spleen (Fig. 2C). In addition, B cells compartment was strongly affected by belatacept at day 0, with a decrease in total CD19 + percentage and fewer plasma cells identified by CD138 and CD38 positive cells (Fig. 2C) only with the treatment at day 0.

**Figure 3:**
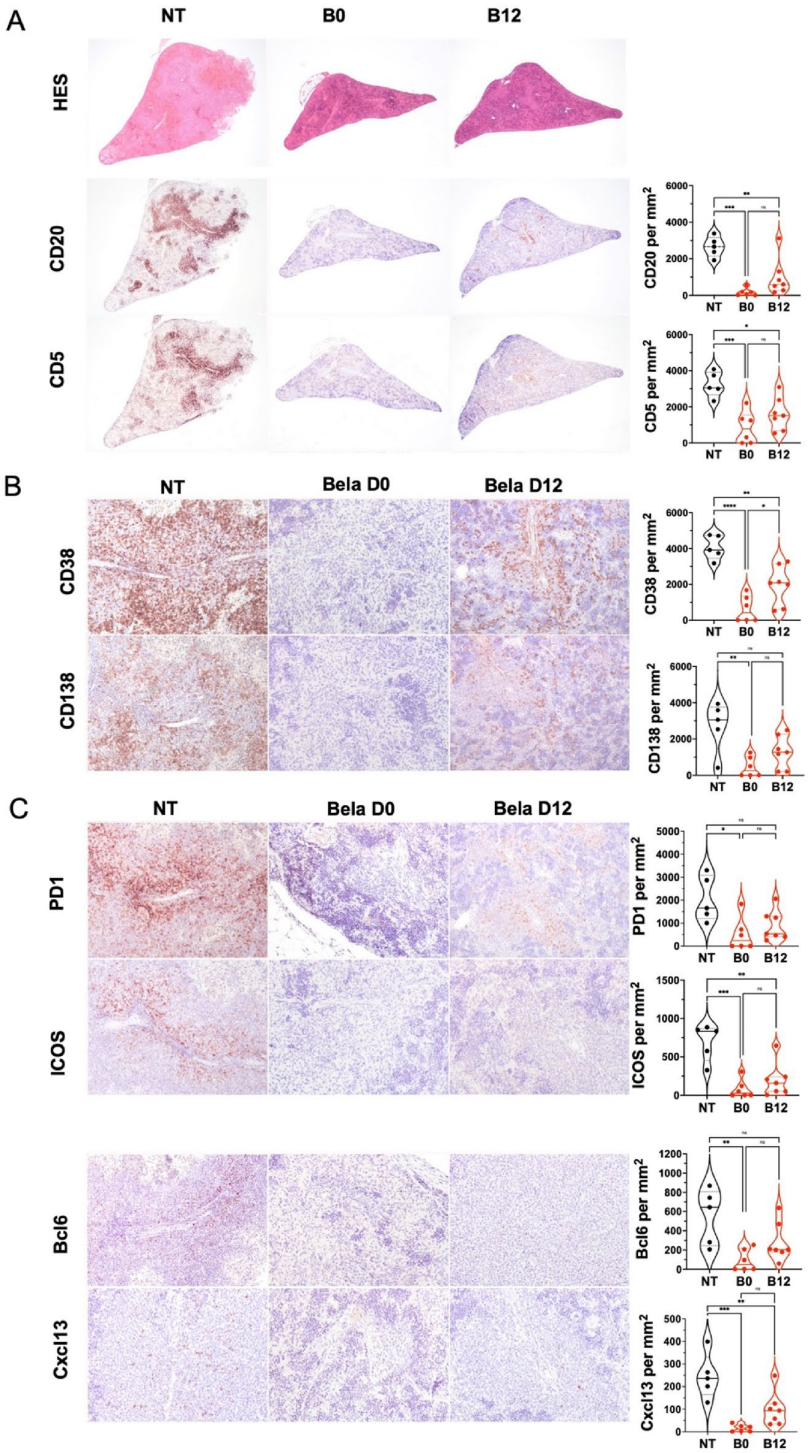
Belatacept inhibited human B and Tfh cells engraftment. Intrasplenic injection of NSG mice were realize with 10 × 10^6^ of CD8 depleted PBMCs (CD8-). Belatacept (5 mg/kg) were injected either at the same day of cells injection (B0) nor at day 12 (B12). Untreated mice (NT) served as control group. Mice were sacrificed at day 21 and spleens were harvested and fixed for immunohistological analysis. (**A**) Serial sections were stained with hemotoxylin and eosin (× 20), anti-human CD20/CD5 (× 20) (**B**) and anti-human CD38/CD138 (**C**) anti-human PD1/ICOS/Bcl6/CXCL13 (× 100 or × 200). Histological quantification of human CD5 + T cells, ICOS + or PD1 + Tfh cells, BCL6 + and CXCL13 + GC cells, CD19 + B cells, CD27 + /CD38 + B differentiated cells and CD138 + cells per mm^2^. The cumulative data of two independent experiments are shown. Each point represents a single xenograft; data are presented as mean percentage ± SEM. Statistical significance from the controls (NT) was determined using one-way anova and Tukey’s multiple comparisons test. *p* ≤ *0.05, **0.01 and ***0.005.

We next analyzed by histology the human GC formation and structure 21 days after the IS infusion of CD8-human PBMCs. In the presence of belatacept, spleens of NSG mice show significantly fewer B zones, both in terms of naive cells (CD20 +) (Fig. 3A) and differentiated cells (CD38 + and CD138 +) (Fig. 3B). We observe a significant reduction in CD5 + T cells from the T cells area, only in the belatacept day 0 group (Fig. 3A). Furthermore, a significant reduction of Tfh markers expression (ICOS, PD1, Bcl6, CXCL13) was observed in the spleens of mice treated with belatacept (Fig. 3C). Belatacept appears to have a specific effect on the development and maintenance of secondary lymphoid structures.

**Figure 4:**
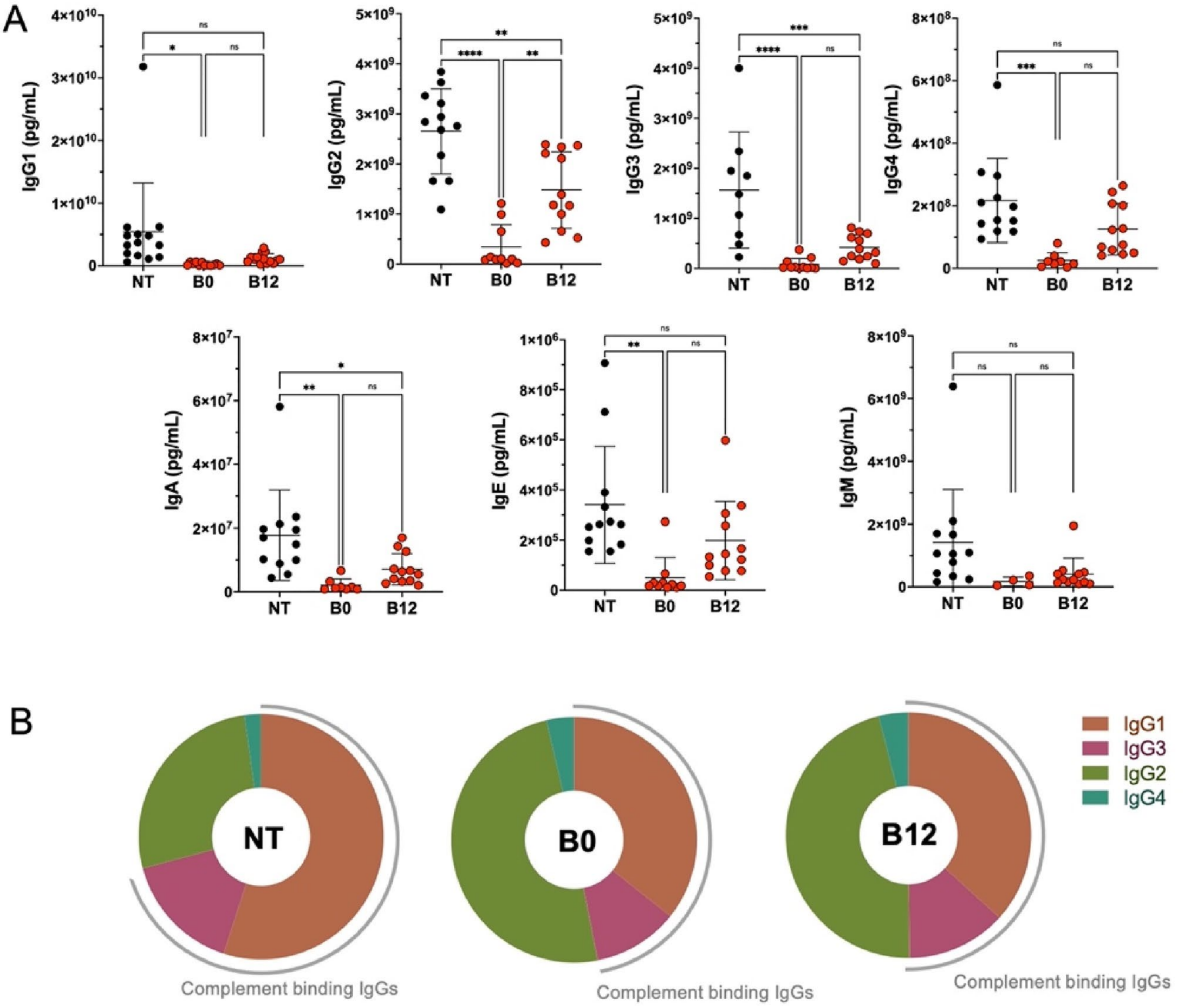
Belatacept treatment affect the plasma cells’ ability to secrete immunoglobulins. Intrasplenic injection of NSG mice were realize with 10 × 10^6^ of CD8 depleted PBMCs (CD8-). Belatacept (5 mg/kg) were injected either at the same day of cells injection (B0) or day 12 (B12) Untreated mice (NT) served as control group. (**A**) Serum of mice were collected at day 21 and human immunoglobulin, IgG (1, 2, 3 and 4), IgA, IgE, and IgM were analysis by Luminex assay. The cumulative data of five independent experiments are shown. Each point represents a single xenograft; data are presented as mean percentage ± SEM. Statistical significance from the controls (NT) was determined using one-way anova test and Tukey’s multiple comparisons test. *p* ≤ *0.05, **0.01, ***0.001 and ****0,0001. (**B**) Pie chart of IgGs repartition between complement binding IgGs (IgG1, IgG3) and non complement binding IgGs (IgG2,IgG4) for the three groups.

We next investigate the functionality of germinal center B cells by analyzing human immunoglobulin production in the sera of mice. Human Ig (IgG1, 2, 3, 4, IgA, IgE, IgM) were detected in large amounts in untreated mice (Fig. 4A). However, belatacept treatment at day 0 and to a lesser extend at day 12 induced a significantly lower level of all IgG subtype tested but also IgA, IgE, and IgM suggesting an altered B cell function in the germinal center. Complement binding IgGs (IgG1 and IgG2) proportion were decreased with belatacept treatment B0 and B12 (Fig. 4B) suggesting a switch in IgG subtype secretion with belatacept.

## Discussion

Controlling the alloantibody response is a key factor for allograft survival. Although this humoral response largely depends on the development of GC in secondary lymphoid organs, this step remains poorly understood and studied in human due to the difficulty of access to these tissues. To date, experimental models allowing analysis of the allospecific immune response within GC response are limited^13,14^. Here we establish an original in vivo model of human GC formation in NSG mice and next demonstrate that belatacept specifically impairs the formation of these GC.

In the field of solid organ transplantation, DSA secretion implies the migration of previously activated B and T cells to the border of B-cell follicles and T-cell zones, where they undergo cognate interactions. At this step, Tfh–B-cell crosstalk leads to the development of the GC and the generation of memory B cells and long-lived plasma cells^15^. Although co-stimulation molecules are fundamental for T-cell priming by dendritic cells they have also been proven to play an important role in the different steps of Tfh differentiation and function during CG formation^16,17^. Indeed, it has recently been found that continuous CD28 expression is required for Tfh differentiation^18^. Along parallel lines, CTLA-4 expression, the inhibitory competitor of CD28, restrains Tfh responses and inhibits their B-cell stimulatory function. Specifically, deletion of CTLA4 on Tfh increased GC B cell numbers and serum antibody titers^19,20^.

Although belatacept has been mainly developed to target DC-priming of T cells, preclinical models have interestingly shown that the use of belatacept inhibited humoral responses in transplantation settings. Follicle size, GC proportion, and IL-21 secretion were decreased in belatacept-treated primate recipients, suggesting a specific role of belatacept on Tfh–B-cell crosstalk^10^. Studies by Chen et al.^21^ and Young et al.^22^ further showed, in murine models, that late CTLA4-Ig administration could inhibit ongoing humoral response, even if priming of allogeneic T cells had already occurred. Indeed, the introduction of CTLA4-Ig treatment 14 days after sensitization inhibited alloantibody production and collapsed GC responses. These results suggested that CTLA4-Ig action on B-cell stimulatory capacities were important, independently of T-cell priming by DCs. Our model offers mechanistic understanding of both clinical and in vitro data suggesting that CTLA4-mediated costimulatory blockade impairs specifically several steps of the humoral response. In fact, belatacept appears to have a specific effect on the development and maintenance of secondary lymphoid structures. We observed a decrease in the size of the follicle and the amount of GC.

We further studied mechanisms underlying belatacept action on humoral response in humans^11^. We have previously show that belatacept inhibited Tfh–B-cell crosstalk in vitro by decreasing the proportion of activated PD-1^+^ICOS^+^ Tfh cells, decreasing Tfh proliferation, and decreasing the differentiation of B cells into plasmablasts. These in vitro data account for clinical results showing a marked reduction in de novo DSA synthesis in renal transplant recipients treated with belatacept when compares to CNI^9^. Here again, the co-stimulatory blockade agents have serious consequences on the quality of GC. It implies that targeting co-stimulatory pathway led to the induction of a new equilibrium of T and B cells population and functions. A significant reduction in Ig production confirms functional consequences of these observations.

With the development of new molecules aiming to control the humoral response in the field of transplantation, it is now essential to have adequate models and tools to evaluate their effectiveness and understand their mechanisms. The model of GC reconstitution described in this work allows to address these two points. It should also allow to test combined approaches associating several therapeutic molecules targeting different cell populations at the same time. Even if this model is not perfect because it does not resume in its integrality what happens in humans, it is to date the model that seems to us the most successful allowing in vivo the study of the B response. This model could be used in the field of alloreactivity as well as in that of autoimmune diseases.

## Methods

### Mice

NSG (NOD/scid/IL-2Rγ-/-) mice were obtained from Charles River (Miserey, France). Manipulations were performed according to European Union guidelines and with approval of the Regional Ethics Committee in Animal Experimentation no. 16, Ile‐de‐France, France (authorization no. 11/12/12‐11B). In vivo experiment reported in this study were done in accordance with ARRIVE guidelines.

### Blood sample

Human PBMCs were obtained from healthy donors (Etablissement Francais du Sang, Créteil, France). Ethical review and approval were not required for the study on human participants in accordance with the local legislation and institutional requirements. The participants provided their written informed consent to participate in this study. PBMCs were isolated by density gradient centrifugation (Lymphocyte Separation Medium; Eurobio®, France). CD8 depletion was made thanks to a negative depletion with CD8 + T Cell Isolation Kit from Miltenyi Biotec (Paris, France).

### In vivo experiment

10 × 10^6^ PBMC (depleted or not in CD8 +) are injected in recipient mice by an intrasplenic injection. Before surgery, mice were given an analgesic solution of buprenorphine. Then, they are kept anesthetized under isoflurane gas. The mice weight and their general state of health (signs of xenoGVHD) are checked 3 times a week.

### Belatacept treatments

Recipient mice were treated by one intraperitoneal injection at day 0 (B0 group) or day 12 (B12 group) of belatacept (Nulojix, Bristol-Myers Squibb Pharma) at 5 mg/kg.

### Flow cytometry

Human cells suspensions from mice spleen and bone marrow were prepared by mechanical dilacerations and then stained for the phenotype analysis. APC/Cyanine7 anti-CD19 (clone HIB19) was purchased from Biolegend (Paris, France). APC anti-CD45 (clone HI30) and PE anti-CD8 were purchased from BD Biosciences (Le Pont de Claix, France). PE-Vio770 anti-CD38 (IB6) was purchased from Miltenyi Biotec (Paris, France). Efluor 506 fixable viability dye was purchased from eBioscience (Paris, France). PE anti-CD138 (clone B-A38) was purchased from Beckman Coulter (Villepinte, France). Events were acquired on a FACS Canto II flow cytometer using FACS Diva software (BD Biosciences), and data were analyzed using FlowJo software (Tree Star, Ashland, OR).

### Immunohistochemistry

For immunoenzymatic staining, spleens were collected. Formalin fixation, paraffin inclusion, as well as standard staining (hematein-eosin) were performed on this tissue. Immunohistochemical staining was performed on 3‐µm–thick tissue sections from the formalin‐fixed paraffin‐embedded spleen specimens, by a standardized automated method (Bond; Leica Menarini) using anti-CD20 M0755 (L26 clone, Dako, anti-CD5, NCL-L-CD5-4C7, 4C7 clone, Leica Biosystems), anti-CD38 (NCL-L-CD38-290, SPC32 clone, Leica Biosystems), anti-CD138 (M7228, MI15 clone, Dako, anti-CXCL13, MABB801, clone 53610, R&D systems), anti-BCL6 (PA0204, LN22 clone, Leica Biosystems), anti-ICOS (ab105227, SP98 clone, Abcam), and anti-PD1 (ab52587, NAT105 clone, Abcam). Slides were scanned using NanoZoomer Digital Pathology System (Nanozoomer 2.0-HT slide scanner (Hamamatsu, Hamamatsu City, Japan)). Cell detection was conducted using QuPath’s digital software (https://qupath.github.io) built-in ‘Positive cell detection’. Cell densities of immune cells were expressed as the mean number of positive cells per mm^2^.

### Luminex

Ig concentrations were measured in the mice serum by Luminex following the manufacturer’s protocol (Affymetrix E-bioscience; Human Isotyping procartaplex). Cytokines were quantified in the mice serum using the human Premixed Multi-Analyte kit from Bio-Techne with Luminex-based technology as specified by manufacturer. The following cytokines were analyzed: IFN-γ, TNF-α and Granzyme B^23^.

### Statistical analysis

Statistical analyses of differences between groups were performed using paired t test or one-way anova follow by Tukey’s multiple comparisons test, with the software Prism 9.0 (Graph Pad Software, Inc., La Jolla, CA). All statistical tests were considered statistically different when *p* < 0.05, *p < 0.05, **p < 0.01, ***p < 0.001, ****p < 0.0001.

### Supplementary Information

Supplementary Figure S1.

## Data Availability

Data are available on reasonable request, please contact corresponding author.
