# Supplementary material for: Belatacept inhibit human B cell germinal center development in immunodeficient mice
Source: Sci Rep. 2023 Aug 24;13:13816. doi: 10.1038/s41598-023-40700-w (PMC10449885; doi:10.1038/s41598-023-40700-w)
Supplement: Supplementary file 1 — Supplementary Figure S1. [file 41598_2023_40700_MOESM1_ESM.docx]

**
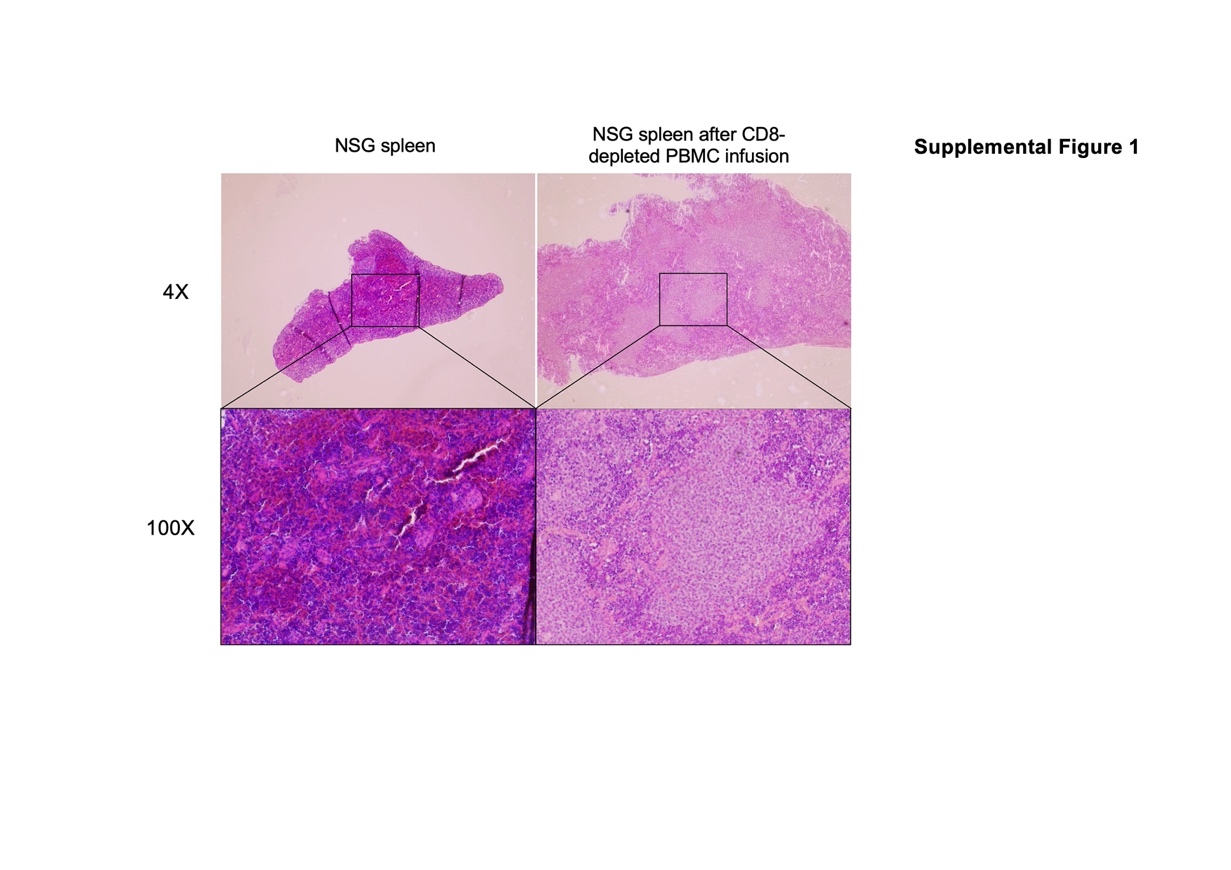
**

**Supplemental figure 1: Comparision between NSG spleen before and after human cells infusion.** Serial sections of normal NSG spleen and CD8-depleted PBMC infused mice at d21 were stained with hemotoxylin and eosin (x4 and x100).
